# Predictors of STD Screening From the Indigenist Stress-Coping Model Among Native Adults With Binge Substance Use

**DOI:** 10.3389/fpubh.2022.829539

**Published:** 2022-08-12

**Authors:** Maya Magarati, Rachel Strom Chambers, Gayane Yenokyan, Summer Rosenstock, Melissa Walls, Anna Slimp, Francene Larzelere, Angelita Lee, Laura Pinal, Lauren Tingey

**Affiliations:** ^1^Seven Directions, A Center for Indigenous Public Health, Department of Psychiatry and Behavioral Sciences, School of Medicine, University of Washington, Seattle, WA, United States; ^2^Department of International Health, Johns Hopkins Center for American Indian Health, Whiteriver, AZ, United States; ^3^Johns Hopkins Biostatistics Center, Baltimore, MD, United States; ^4^Department of International Health, John Hopkins Center for American Indian Health, Great Lakes Hub, Duluth, MN, United States

**Keywords:** sexually transmitted diseases (STD), cultural buffering, enculturation, historical loss, stress coping and/or resiliency, perceived discrimination, American Indian/Alaska Native, sexual and reproductive health service

## Abstract

**Objective:**

The American Indian/Alaska Native (AI/AN) population in the U.S. is thriving in spite of settler colonialist efforts of erasure. AI/AN people, however, continue to experience persistent health disparities including a disproportionate burden of substance use and sexually transmitted diseases/infections (STDs/STIs), as well as a disproportionate lack of public health STD screening services and STD prevention interventions grounded in AI/AN social contexts, experiences, and epistemologies. The present study explored how stressors and protective factors based on the Indigenist Stress Coping framework predict STD screening outcomes among Native adults.

**Methods:**

We analyzed baseline self-report data from 254 Native adults ages 18–55 years with recent binge substance use who were enrolled in an evaluation of “EMPWR,” a two-session STD risk reduction program in a rural, reservation-based community in the U.S. Southwest. Logistic regression models with robust variance were used to estimate odds ratios of lifetime STD testing for the theoretical stressors and cultural buffers.

**Results:**

A little over half the sample were males (52.5%, *n* = 136), with a mean age of 33.6 years (SD = 8.8). The majority (76.7%, *n* = 195) reported having ever been screened for STD in their life. Discrimination score were significantly associated with lifetime STD testing: The higher discrimination was associated with lower odds of STD testing in the fully adjusted model (aOR = 0.40, 95%CI: 0.18, 0.92). The effects of AI/AN-specific cultural buffer such as participation in traditional practices on STD testing outcomes was in the expected positive direction, even though the association was not statistically significant. Household size was significantly associated with STD screening: The higher the number of people lived together in the house, the higher the odds of STD testing in the fully adjusted model (aOR = 1.19, 95%CI: 1.04, 1.38).

**Conclusion:**

Our findings suggest that STD prevention programs should take into consideration AI/AN-specific historical traumatic stressors such as lifetime discrimination encounters and how these interact to drive or discourage sexual health services at local clinics. In addition, larger household size may be a protective factor functioning as a form of social support, and the extended family's role should be taken into consideration. Future research should consider improvement in measurements of AI/AN enculturation constructs.

## Introduction

The American Indian and Alaska Native (AI/AN) population in the United States is growing and thriving in spite of settler colonialist efforts of erasure. Recent census data show that from 2010 to 2020, the AI/AN-alone population grew by 27.1%, and AI/ANs in combination with other race/ethnic subgroups (or AI/AN mixed ethnicity) grew by 160% (1). AI/ANs, however, continue to experience persistent health inequities including a disproportionate burden of sexually transmitted diseases (STDs), also referred to as sexually transmitted infections (STIs). For the purposes of this manuscript, we use STD interchangeably with STI. While recent national surveillance data between 2016 and 2019 show annual increases in all three common reportable STDs across races/ethnicities, i.e., chlamydia (+19% since 2015), gonorrhea (+56%), primary and secondary syphilis (+74%), as well as congenital syphilis (+279%), STD rates are disproportionately higher among AI/ANs compared to Whites and all other racial/ethnic groups except for Blacks (2, 3). In 2019, compared with Whites, the chlamydia rate among AI/ANs was 3.6 times greater (760 vs. 209.7 per 100,000, respectively), gonorrhea was 4.8 times greater (355.8 vs. 73.9 per 100,000), and primary and secondary syphilis was 3.2 times greater (21.3 vs. 6.6 per 100,000) (2).

Given the high rates of STDs in the AI/AN population, and the serious health consequences of STDs if left untreated, AI/ANs are deemed a priority population (2) for STD intervention including testing and screening for STD, and promotion of sexual health equity (3, 4). STD transmission probability is influenced by various individual behaviors such as condom use, number of new partners per unit of time, sexual partnering, community STD prevalence, and syndemics such as substance use and hepatitis C virus (HCV) infections (3). In addition, the complex structural and social contexts of historical and ongoing oppression and resulting exposure to stressors and trauma within which these factors interact also drive inequities in STD rates among AI/ANs (5).

AI/ANs also experience disproportionately high rates of alcohol and drug use and comorbid substance use disorders which are associated with increased STD risk. In 2020, 9.1% of Native adults reported past month heavy drinking and 14% reported past year Alcohol Use Disorder, the highest rates of any race/ethnic group, while in 2019, 22.8% of Native adults reported past month binge alcohol drinking (6). Further, 25.5% of Native adults reported having a substance use disorder in the past year, more than 10 percentage point above the rate for Whites. More specifically, alcohol and other substance use are associated with increased STD risk through unsafe sexual practices including unprotected sex (7), as well as increased number and concurrency of sex partners (8). Research conducted with AI/ANs who engaged in sex while drunk or high showed they were 14 times more likely to engage in sexual risk behaviors than those who had not (9). Screening for STD among AI/ANs who binge substance use is particularly important.

While data on screening rates of Native adults is sparse, there is ample information about structural determinants that preclude STD testing in Native communities including a shortage of sexual healthcare providers and access barriers to healthcare including confidentiality concerns, and geographic isolation (10–15). In relation to access to STD testing among participants in this study, in the community in which this study was conducted which spans over 1.5 million acres, there is only one health care facility in which STD testing is available and STD testing by mail was not offered. Community members often travel 60 miles or more to access services at the one clinic.

An important contextual barrier specific to AI/AN healthcare access is that rural and/or reservation-based areas have historically had limited and/or underfunded medical and social services that limit the delivery and quality of STD care. Furthermore, among AI/ANs residing in these communities, stigma, provider bias, and mistrust of the health care system contribute to decreased health care utilization resulting in delays in diagnosis and treatment, which pose significant challenges to STD prevention efforts (3). In small tribal communities on reservations where personal relationships exist between health care seeking community members and health care providers, fear of potential breach of confidentiality in STD testing and treatment are additional barriers (16, 17).

The Centers for Disease Control and Prevention's newly released STD treatment guidelines indicate that primary prevention of STDs should include assessment of both behavioral risk (i.e., assessing the sexual behaviors that can place persons at risk for infection) and biologic risk (i.e., testing for risk markers for STD and HIV acquisition or transmission) (18). In addition, the National Academies of Sciences, Engineering, and Medicine (NASEM) articulates that efforts are needed to determine how to best integrate health services for populations that are at risk of substance use and syndemic infections, such as HIV and STDs in order to provide integrated care delivery models for STDs and related syndemics (3). This paper focuses on STD screening among Native adults with binge substance use who reside on a rural reservation.

### Conceptual Framework

Several tribes and Tribal Epidemiology Centers in partnership with universities have developed culturally relevant and age-appropriate tribal best practices of sexual health curricula for AI/AN youth, including several by the tribal-academic team who co-authored this manuscript (16, 19, 20). Classical theories of behavioral change—in particular, risk perception and behavior intention—have been found helpful in explaining individual-level mechanisms of sexual risk or protective behaviors among AI/ANs. For example, lower perception of risk to STD susceptibility, generally due to misconceptions about STD prevention and treatment, have been found to be associated with risky sexual behaviors among AIs (21, 22). Alternatively, in Native communities it is important to consider the importance of communal risk; literature specific to Native communities underscores Native cultural practices and values about the importance of family and community for promoting health and wellness (23, 24). Thus, we suggest taking a more holistic view of behavior change when working with Native communities which includes utilizing Native-centered theories and including measures such as household size as a proxy measure for social support and family engagement (25). Furthermore, treatment approaches that are congruent with AI/AN cultural values, traditions, and customs enhance treatment engagement (26–28).

A number of culturally relevant organizing conceptual frameworks have emerged in the last two decades that synthesize our understanding of and ways to address health inequities among AI/ANs (29). These include the Native-Reliance theoretical framework to explain substance use in AI/AN youth, the Indigenist Stress-Coping (ISC, a stress process approach) model to explain health outcomes including STD/HIV infections among other health conditions (5, 30, 31) and the Framework of Historical Oppression, Resilience, and Transcendence (FHORT) which has been used to explain violence (32, 33) and depression experienced by Indigenous peoples (33). The contributions of these models and others suggest that Native cultural values, identities, and traditional and spiritual practices are critical for AI/AN peoples to cope with stressors and thrive for wellbeing. For example, enculturation—inclusive of positive identification with AI/AN ethnicity, internalization of cultural values, connectedness to and participation in community and traditional cultural practices, and participation in spiritual traditions—has been found to protect AI/ANs against risks for STDs (34–37). Cultural identity manifested as social support and protective family and peer influences have also been cited as protective buffers for substance use and risky behaviors among AI youth (38). Thus, it is imperative to consider cultural tailoring processes in substance use interventions including incorporating traditional AI/AN rituals, practices, ceremonies, and cultural activities (e.g., talking circles) (39).

This paper aims to apply the Indigenist Stress-Coping (ISC) theoretical framework to examine Indigenous-specific risk and protective predictors of STD screening among rural, reservation-dwelling AI adults who binge drink or use drugs (30, 31, 40). The ISC is a stress process model of understanding health disparities, that emphasizes how socio-cultural and historical processes influence stress exposure and the impact of stress on AI/AN individual behavioral health. Stressors may stem from a complex interplay of contemporary structural and interpersonal discrimination and violence exposure, socio-economic and environmental structural inequities, and, historical and lifetime cultural trauma rooted in histories and legacies of settler colonialism, forced relocation and removal from land (41, 42). These stressors have been associated with high risk-taking behaviors. Stressful life events in AI youth have been shown to be positively associated with substance use and risky behavior (38). Similarly, traumatic life events and stress have been associated with sexual risk behaviors in AI youth and adults. This was especially the case among young women in a study conducted with a Northern Plains tribe (43) and in a study with a Southwestern tribe (7, 25).

**Figure 1:**
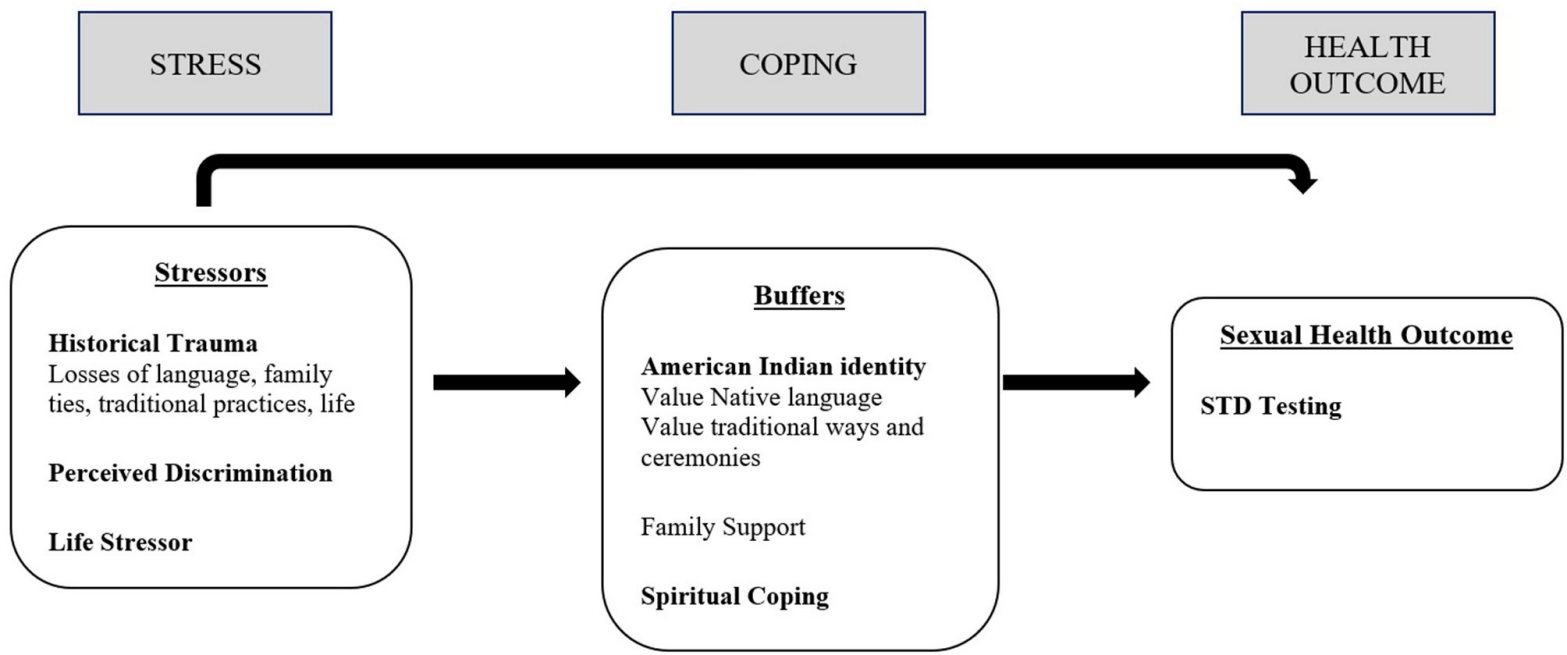
Modeling indigenist stress-coping model predicting STD testing. Adapted from Walters and Simoni (31) and Walters et al. (5, 44, 45).

We will examine the factors that predict STD screening outcomes in a high-risk population of rural, reservation-dwelling AI adults who binge drink or use other substances. AI people who reside on tribal lands tend to have strong connections to their ancestral geographies, original instructions, traditional cultural practices, and ways of life including reciprocal relationality grounded in family and community support (44). The ISC model proposes that AI/ANs' sexual health outcomes should be understood in the context of their unique socio-demographic vulnerabilities due to historical and current trauma that give rise to multiple co-occurring stressors (see Figure 1). The ISC framework articulates that Indigenous cultural, community, spiritual, and personal resources/strengths may buffer the effects of stressors and risk on poor sexual health outcomes (31).

Historical social events such as historical trauma (HT) (e.g., collective loss or pain) that interact with proximal contemporary chronic stressors such as microaggressions, discrimination, exposures to lifetime trauma, and interpersonal violence are unique to AI/AN-specific contexts and may impact healing, health, wellbeing, and stress processes (44, 46). At the individual level, HT impact includes impairments in family system disruption including communications (47), mental health symptoms, substance abuse (46), and sexual risk behaviors (43, 44). At the community level, collective responses to HT include the disruption of Native culture such as Native identity, languages, traditional practices, and spirituality (44, 48).

The Historical Loss Scale (HLS) is a standardized measure that assesses the frequency with which AI/ANs think about the losses to their culture, land, and people as a result of settler colonization (41, 46). The HLS is conceptualized as a contributor to negative contemporary experiences of historical trauma or historical grief, and thus a stressor (41). These persistent thoughts of historical loss appear to have emotional and behavioral consequences. They are associated with alcohol abuse, anger, and symptoms of internalization and violence (46). Researchers posit that the more an AI/AN individual thinks of the collective losses and associated griefs rooted in historical injustices endured by their communities, the higher the level and intensity of contemporary stress they will experience (41, 44, 45, 49).

Another stressor in the ISC framework is perceived or experienced discrimination, by which AI/ANs experience negative or unfair treatment because of their ethnicity. Interpersonal discrimination can affect health and health-seeking behaviors (50, 51). Research has shown that perceived and experienced interpersonal discrimination against AI/ANs and other social determinants described above produce heightened stress responses, affect risky health behaviors (40, 50, 51), and impede equitable access to sexual healthcare services including regular STD screening, care, and treatment.

Cultural factors have been found to buffer AI/ANs against the negative health effect of stressors. AI/AN enculturation is the process by which individuals learn about and identify with their ethnic minority culture (increasing knowledge about, engaging with, and identifying with traditional cultural practices). Across studies, enculturation has been found to be protective in nature, promoting academic success and decreasing violent behavior and alcohol abuse among AI youth (41, 52). Specific aspects of enculturation including connection to traditional cultural values and ethnic identity and a sense of family belongingness have been found to be protective against heavy alcohol use (52).

Enculturation may hold a particular strength for AI/ANs who were born and raised in the decades since the 1960's and 1970's tribal self-determination movement era “when tribes were able to “determine” their identity, or in other words, to create their own identity through defining and affirming their cultural values” (53), p. 782. Although the Civil Rights movement of the 1960's brought high hopes for social and racial justice within the U.S., institutional and interpersonal racism and discrimination continue to plague the country impacting health disparities (54).

The purpose of this analysis was to explore the predicted Indigenous stressors and cultural buffers of STD screening among AIs with recent binge alcohol use using the ISC model. Thus, we test the following two hypotheses:

**Hypothesis 1**: Stressor exposures such as economic stress, thoughts of historical loss, and perceived or experienced discrimination decrease protective sexual health behaviors such as STD screening.
**Hypothesis 2**: Cultural buffers, namely AI enculturation factors such as Native language, traditional practices and ceremonies, and spirituality buffer against the negative effects of stressors on STD screening.

## Materials and Methods

### Data Source and Participants

#### Study Design and Setting

Data for this paper were gathered in 2015 at the baseline assessment as part of a randomized controlled trial (RCT) evaluating the EMPWR (Educate, Motivate, Protect, Wellness and Respect) intervention for impacts on several sexual health risks, including completion of STD screening (25, 28, 55, 56). EMPWR was culturally adapted from an evidence-based intervention “Project RESPECT” (28). The RCT was developed in the context of a well-established sustainable collaboration between the participating reservation-based tribal community in the Southwestern United States and the study's academic partners. The participating community is located in one of the hot-spot counties in the Southwestern U.S. with higher multi-STD concentration rates than in other counties (2–4, 12, 57). When spatial disparity in STDs juxtaposes on racial disparity, hot-spot analyses indicate that a 10-percentage point increase in the proportion of AIs as a share of the county's population was associated with a 53% increase in the odds of being a hot-spot county for chlamydia and a 55% increase for gonorrhea (57).

The EMPWR program is a brief, client-focused counseling intervention that aims to motivate STD screening and reduce sexual risk-taking behaviors. The study was conducted from July 2015 to June 2019, and the study aims were evaluated at three time points (baseline, 3-, and 6- months post-intervention). Details about the RCT design, protocol and intervention outcomes are reported elsewhere (25, 27, 28, 55). This paper utilizes the cross-sectional baseline data collected prior to participants receiving EMPWR intervention sessions.

The governing Tribal Council and Health Advisory Board of the participating tribe, along with the participating academic and Indian Health Service Area research review boards, approved the study. This article was reviewed and approved by the Tribal Council and Health Advisory Board of the participating tribal community.

#### Participants

Participant inclusion criteria included adults ages 18–55 years who self-identified as AI, were residing in the participating tribal community, were sexually active in the last 3 months (self-report on eligibility screener), reporting at least one episode of binge alcohol or other substance use within the last 3 months, and provided informed consent. Participants were referred for study recruitment from the tribe's public health surveillance system which tracks all incidents of binge substance use, defined as use which results in serious consequences associated with a high blood alcohol level or drug toxicity level (27, 28). Surveillance staff are authorized by tribal law to conduct in-person follow-up visits with every reported individual to confirm the event and triage the individual to outpatient treatment (27, 28). During that visit, surveillance staff screened potential participants for study eligibility criteria. All participants were consented by trained local study staff and then completed a baseline assessment. In total, 301 participants were consented and completed the baseline assessment.

#### Cultural and Contextual Adaptation

Tribal community stakeholders engaged in decision making in all phases of study design. One of the strengths of the research partnership is that local para-health professionals served on the research team as local research staff. All evaluation instruments including the baseline assessment questions were adapted and piloted with members of the participating tribal community. For instance, the “loss of land” item was dropped from the standard HLS instrument for this community. In order to meet the transportation needs of the participants and assure confidentiality in a small, rural, close-knit tribal community, baseline self-report surveys were completed *via* Audio Computer Assisted Self-Interview technology on laptops at participants' own homes, in the research team's local study office, or in a mutually convenient location in the community, including inside project vehicles (27).

### Measures

All measures in these analyses were assessed via self-report survey responses and include sociodemographic characteristics, stressors, and cultural and spiritual buffers, as well as protective and risky sexual health behaviors. Participants received $15 USD for completing self-report baseline assessments via Audio Computer Assisted Self-Interview technology on laptops, or via hard copy.

#### Outcome Measure

The primary outcome variable used in the analysis in this paper is, “Ever screened for an STD,” which was measured by one question which asked if participants had ever been tested for an STD. Response options included: Yes, No, I don't know, or Refuse to answer. For the purposes of this analysis, we dichotomized the outcome variables (0 = no and 1 = yes). The “I don't know” or “Refuse to answer” responses were coded into missing. Participants with missing values for STD screening were removed from the analytic dataset.

#### Control Variables

Sociodemographic characteristics such as age (18–55 years), sex (male = 0, female = 1), has children (none = 0, has any number of children = 1), marital status (single = 0, married or cohabiting = 1), education (less than high school = 0, high school, GED or above = 1) and number of people who live together (count) as an indicator of household size (a proxy for social support) were each assessed via self-report survey responses.

#### Stressors

Table 1 presents stressors and cultural buffers, and corresponding Cronbach's alpha for scales. Three measures of psychosocial stressor exposure were included in these analyses: Economic stress, Historical Loss Scale (HLS), and the Perceived Discrimination Scale.

**Table 1:** Measures of stressors and cultural buffers operationalized for indigenist stress coping (ISC) model among American Indians with binge substance use in a rural community, *N* = 254.

**Variable**	**Number of items**	**Response options**	**Mean (SD)**	**Alpha**
**Life stressors**				
***Economic stressor***				
Do you currently have a job?	1	Yes (0); no (1)	0.09 (0.29)	N/A
**Traumatic stressors**				
***Historical loss scale***	10	How frequently do the following losses come to mind? Never (0); yearly or special times (1); monthly (2); weekly (3); daily (4); several times a day (5).	1.41 (1.36)	0.924
Fewer people speaking our language				
Less knowledge and participation in our traditional and spiritual ways				
Less knowledge and participation in cultural activities				
Less understanding in preparing Traditional healthy foods and activities				
The effects of alcoholism on our people				
Loss of our people through early death (alcohol related, suicide, accidental, etc.)				
Loss of respect by our children and grandchiildren for elders				
Loss of family ties due to Boarding/residential schools				
The loss of self-respect from poor treatment by government officials				
The loss of trust in white people from broken treaties				
***Perceived discrimination scale***				
*Global discrimination sub-scale*	5	How often have you experienced the following? Never (0); sometimes (1); always (2).	0.49 (0.53)	0.915
Someone said something bad or insulting to you because you are Native American				
Someone ignored you or excluded you from some activities because you are Native American				
Someone said a racial slur or racial insult to you or about you				
You felt threatened by someone because you were worried that they may harm you because you are Native American				
Someone treated you unfairly because you are Native American				
**Cultural buffers**				
***American Indian identity (enculturation)***				
***Use of native language***	1		0.52 (0.50)	N/A
What language do you prefer to speak? (Only choose one answer)		Your tribe's language (1); English (0); both English and your tribe's language (3); other (4); I don't know (97); refuse to answer (98).		
***Practice of traditional ceremonies and ways***	1	Not at all (0); not very important (1); somewhat important (2); very important (3); I don't know (97); refuse to answer (98).	1.57 (1.14)	N/A
How important is it to you to participate in your tribe's Traditional practices?				
***Spiritual coping scale***				
To what extent is your spirituality involved in understanding or dealing with stressful situations in any way?	1	Not involved at all (0); not very involved (1); somewhat involved; (2) very involved (3); I don't know (97); refuse to answer (98).	1.46 (1.06)	N/A

Economic stress was measured by whether the participant was unemployed at the time of the baseline assessment (0 = employed, 1 = unemployed).

The HLS is a 10-item standardized measure. The participants were asked to report the frequency with which they think about the three types of losses due to the historical process of colonization: the losses to their culture, land, and their people, as well as thoughts of mistreatment for being AI (46). Two items specific to loss of land and loss of families from the reservation due to government relocation to reservations and urban areas were deemed inapplicable to the participating tribal community's context, and were excluded. Average of non-missing responses to 10 items on a Likert scale was used to assess thoughts of historical loss (possible range: 0 = never, 1 = yearly or at special times, 2 = monthly, 3 = weekly, 4 = daily, and 5 = several times a day) so that higher values indicate more frequent thoughts about historical loss. The ten-item HLS had high internal consistency (Cronbach's alpha coefficient = 0.924).

For STD screening outcome analysis, the HLS was categorized as <3, 3 or greater and missing, which includes participants with missing data to all 10 items. Three subscales were also derived from the HLS per previous research (41, 46, 58): loss of culture (alpha = 0.802); loss of loss of early life due to alcoholism or other accidents (alpha = 0.859); and thoughts of cultural mistreatment in terms of loss of respect, self-respect, trust, and family ties due to colonization (alpha = 0.878). Since the ten-item HLS performed better than each of the sub-scale predictive associations, and fit the model best with the STD screening outcome variable, the overall HLS was selected for final analysis.

Perceived discrimination was assessed with a standard ten-item scale measuring a range of potential types and sources of discrimination (32). The participants were asked to report the number of times they had experienced discrimination defined as negative or unfair treatment because of their ethnicity. Responses to these items were coded so that higher values indicated higher level of perceived discrimination (possible range: 0 = never, 1 = sometimes, and 2 = always). The perceived discrimination scale had high internal consistency (Cronbach's alpha coefficient = 0.949). Average scores across relevant items were used to assess three subscales or dimensions of discrimination. Perceived global discrimination (five items regarding being ignored, excluded, or verbally insulted by others to hearing racial slurs and threats of physical harm), authority discrimination (three items regarding perceived discrimination by authority figures such as police) and school or work discrimination (two items regarding teachers of employers expecting them not do well because they are AI). Exploratory analysis revealed that the five-item global discrimination subscale (Cronbach's alpha coefficient = 0.915) was found to have the strongest predictive association and fit the model best with the outcome variable (the lowest AIC or Akaike Information Criterion), and was selected for the analysis.

#### Cultural Buffers

The self-report survey assessed a variety of cultural affinity and enculturation measures through 17 different questions such as the use of and preference for Native language, traditional cultural values and beliefs, and identification with and involvement in traditional ceremonies and cultural practices. Beliefs and values based on religion and religious practices also constituted this set of theorized buffers. The 17 questions, however, varied in enculturation dimensions, response types, and missing values inhibiting the development of one single enculturation scale or meaningful sub-scales. Therefore, we operationalized enculturation by selecting the two most robust items with the least missing values for analysis, and an entirely separate Spiritual Coping Scale (see Table 1).

As illustrated in Table 1, one measure of AI enculturation pertaining to the transmission of cultural knowledge was a question about participants' preference to speak their Native language in their daily lives. The response categories were *(prefers to speak: 0* = *Native language, 1* = *English, 2* = *Both Native language and English, 97* = *don't know, 98* = *refuse to answer)*.

The second measure of AI enculturation includes the importance respondents placed on participating in traditional practices and ceremonies (How important is it to you to participate in your tribe's traditional practices?). Responses were treated as categories rather than scales for analyses *(0* = *not at all important, 1* = *not very important, 2* = *somewhat important, 3* = *very important, 97* = *don't know, 98* = *refuse to answer*).

Finally, spirituality as a stress coping mechanism was assessed with a one-item Spiritual Coping Scale question, “To what extent is your spirituality involved in understanding or dealing with stressful situations in any way?” Responses were *(0* = *not involved at all, 1*= *not very involved, 2* = *somewhat involved, 3* = *very involved, 97* = *don't know, 98* = *refuse to answer)*.

### Analysis

“Don't know” or “refuse to answer” choices or missing values were coded as separate response categories in the analyses to avoid sample size reduction in the models. Only one numeric variable in the models still has missing data: number of persons living in the household (*n* = 14). Other categorical variables with missing data include education (*n* = 2), having children (*n* = 1) and employment status (*n* = 2). Since the number of missing observations is too small, we couldn't add “missing” as an additional category for these variables. This resulted in inclusion of *n* = 236 participants in our models (*n* = 93% of eligible participants). We also compared characteristics between participants who are included in the analyses and those who do not have a response to ever screened for STD, i.e., with missing data on the STD testing status to assess for any systematic differences. Compared to excluded participants, those included in the analyses were more likely to be married or cohabitating (37 vs. 19%), more likely to prefer to speak English (48 vs. 19%), had higher global discrimination score (median = 0.4 vs. 0), and had somewhat or high extent of spiritual involvement when dealing with stressful situations (41 vs. 9%).

Bivariate associations between sociodemographic variables, theoretical stressors, cultural buffers, and the outcome variable were explored using *t*-tests or Wilcoxon tests for continuous variables, and Pearson's chi-squared tests for categorical variables, except for the situations where the counts in the contingency table were low. In these cases, we used the Fisher's exact test.

Logistic regression models with robust variance were used to estimate odds ratios of the study outcome for the covariates. Visual displays were used to explore potential non-linear relationships between continuous variables, such as age, and the outcome on logit scale. When evidence of non-linear relationship was observed, these were modeled using linear splines with appropriate knots that correspond to change in linear slope.

Models were fit sequentially starting with control variables, and adding measures of economic stress, historical loss, discrimination, and cultural buffers. Akaike Information Criterion (AIC) was used to compare models (including models with different subscales for the HLS). The models with smallest AIC are presented in the results section. Five separate models are shown: economic stress with control variables (model 1), model 1 variables + historical loss scale (model 2), model 2 variables + global discrimination scale (model 3), model 3 variables + preferred spoke language (model 4) and model 4 + importance of participating in traditional practices and spiritual coping scale (model 5).

STATA 16.1 was used to fit the models (58).

## Results

Among the 301 participants who completed the EMPWR baseline assessment, 254 (84.3%) responded to the “Ever screened for STD” question and were included in the bivariate analysis. Due to missing data in the covariates, the multivariate analysis included 236 participants (*n* = 93% of eligible participants). The majority of the individuals reported having ever been screened for STD in their life (76.7%) while 23.3% reported never been screened for STDs (Table 2). Participants on average were 33.6 years old (SD 8.8), and a little over half (53.5%) were males. The majority were single (60.6%) while 39.4% were married or cohabitating, and three-quarters had children (75%). A little over half (52.8%) had completed high school or GED. There were no significant sociodemographic differences between those who had never screened for STD and those who had except for the average number of people living together (*p* = 0.028). On average, individuals who had screened for STD lived with more people (median = 4, IQR = 2–7) than those who had never screened (median = 3, IQR = 2–5).

**Table 2:** Demographic characteristics and select stressors and cultural buffers among American Indians with binge substance use by lifetime STD screening in a rural community, *N* = 254.

	**Total non-missing**	**Ever tested for STD**			
**Variable**	**(*N* = 254)**	**Yes (*n* = 195, 76.7%)**	**No (*n* = 59, 23.2%)**	***p-value***	**Test**
**Sociodemographic Characteristics**					
**Age**	33.6 (8.8)	33.82 (8.67)	32.78 (9.09)	0.43	Two sample *t* test
**Sex**					
Male	136 (53.5%)	101 (51.8%)	35 (59.3%)	0.31	Pearson's chi-squared
Female	118 (46.5%)	94 (48.2%)	24 (40.7%)		
**Marital status**					
Single/divorced/separated	143 (56.3%)	113 (57.9%)	30 (50.8%)	0.39	Pearson's chi-squared
Married/cohabiting	93 (36.6%)	69 (35.4%)	24 (40.7%)		
Missing	18 (7.1%)	13 (6.7%)	5 (8.5%)		
**Have any children**					
No	63 (24.8%)	43 (22.1%)	20 (33.9%)	0.068	Pearson's chi-squared
Yes	190 (74.8%)	151 (77.4%)	39 (66.1%)		
Missing	1 (0.4%)	1 (0.5%)	0 (0.0%)		
**Education status**					
Less than high school	52.1% (135)	87 (44.6%)	32 (54.2%)	0.17	Pearson's chi-squared
High school/GED and above	46.8% (119)	107 (54.9%)	26 (44.1%)		
Missing	2 (0.8%)	1 (0.5%)	1 (1.7%)		
**Household size**					
N of people living in the house	4.5 (8.8)	4.67 (2.80)	3.74 (2.35)	0.028^*^	Two sample *t* test
N of people living in the house	4 (2–6)	4 (2–7)	3 (2–5)	0.032^*^	Wilcoxon rank-sum
**Life stressors**					
**Economic stressor: current employment**					
Unemployed	228 (89.8%)	176 (90.3%)	52 (88.1%)	0.81	Pearson's chi-squared
Employed	24 (9.4%)	18 (9.2%)	6 (10.2%)		
Missing	2 (0.8%)	1 (0.5%)	1 (1.7%)		
**Traumatic stressors**					
**Historical loss scale**					
Score <3	(202) (82.8%)	156 (80.0%)	46 (78.0%)	0.43	Pearson's chi-squared
Score ≥3	(42) (17.2%)	33 (16.9%)	9 (15.3%)		
Missing	10 (0.4%)	6 (3.1%)	4 (6.8%)		
**Global discrimination scale**	0.50 (0.50)	0.47 (0.50)	0.60 (0.61)	0.10	Two sample *t* test
Score <1	185 (72.8%)	147 (75.4%)	38 (64.4%)	0.14	Pearson's chi-squared
Score ≥1	62 (24.4%)	42 (21.5%)	20 (33.9%)		
Missing	7 (2.8%)	6 (3.1%)	1 (1.7%)		
**Cultural buffers**					
**Preferred language spoken**					
Native language	50 (19.7%)	36 (18.5%)	14 (23.7%)	0.92	Fisher's exact
English	121 (47.6%)	94 (48.2%)	27 (45.8%)		
Both English and Native language	72 (28.3%)	56 (28.7%)	16 (27.1%)		
Other	1 (0.4%)	1 (0.5%)	0 (0.0%)		
Don't know	2 (0.8%)	2 (1.0%)	0 (0.0%)		
Refuse to answer	8 (3.1%)	6 (3.1%)	2 (3.4%)		
**Importance of participating in tribe's traditional practices and ceremonies**					
Not at all important	58 (22.8%)	41 (21.0%)	17 (28.8%)	0.90	Fisher's exact
Not very important	42 (16.5%)	33 (16.9%)	9 (15.3%)		
Somewhat important	67 (26.4%)	53 (27.2%)	14 (23.7%)		
Very important	61 (24.0%)	47 (24.1%)	14 (23.7%)		
Don't know	2 (0.8%)	2 (1.0%)	0 (0.0%)		
Refuse to answer	24 (9.4%)	19 (9.7%)	5 (8.5%)		
**Spiritual coping scale: extent to which spirituality is involved in dealing with stressful situations**					
Not involved at all	53 (20.9%)	41 (21.0%)	12 (20.3%)	0.091	Fisher's exact
Not very involved	27 (10.6%)	20 (10.3%)	7 (11.9%)		
Somewhat involved	72 (28.3%)	62 (31.8%)	10 (16.9%)		
Very involved	33 (13.0%)	27 (13.8%)	6 (10.2%)		
Don't know	28 (11.0%)	18 (9.2%)	10 (16.9%)		
Refuse to answer	41 (16.1%)	27 (13.8%)	14 (23.7%)		

* *p < 0.05*.

*Data are presented as mean (SD) or median (IQR) for continuous measures, and n (%) for categorical measures*.

### Bivariate Associations: Life and Traumatic Stressors

The majority of the participants (89.8%) were not employed at the time of the survey, indicating a high level of economic stress. There was however no significant difference in this stressor between those who have ever screened for STD and those who had not (90.3 vs. 88.1%, *p* = 0.81).

Overall, the majority of the participants (82.8%) reported that they either never thought about historical losses due to colonization, and if they did, the majority only thought about it once a year or on special occasions. Only 17.2% of the participants reported thinking about the losses weekly, daily, or several times a day. The general low frequency of historical loss thoughts was reported by both those who had ever screened for STDs in their life and those who had not (16.9 vs. 15.3%, *p* = 0.43).

In general, participants reported having never or rarely experiencing discrimination due to having Native American identity, whether it was global, authority or school/work discrimination. Lack of or low level of global discrimination was similar between those who had ever screened for STD and those who had not (mean = 0.47 vs. 0.60, *p* = 0.10).

### Bivariate Associations: Cultural and Spiritual Buffers

Associations of AI enculturation and spiritual coping measures with lifetime STD testing are indicated in Table 2. Half of the participants (50.2%) preferred to speak either their Native language or a combination of Native language and English in their daily lives, and about half (49.8%) preferred to speak English only. Preference for speaking their Native language did not differ between those who had screened for STD and those who had not (18.5 vs. 23.7%, *p* = 0.92).

In regards to actual participation in tribal ceremonies and traditional practices, 24% of participants believed it was very important to participate in their traditional ceremonies, and another 26% reported it was somewhat important, while 39.4% expressed that it was either not at all important or not very important. Ten percent of participants either said “don't know” or refused to answer to this question. There were no significant differences in these beliefs between those who had and had not been previously screened for STD (*p* = 0.90).

Finally, 41% expressed that their spirituality was somewhat involved or very involved in helping them understand or deal with stressful situations. However, 16% of respondents refused to answer this question. The level of spirituality's involvement in stress coping did not differ significantly between those who had ever tested for STD and those who had not (*p* = 0.09).

### Multivariate Relationships With STD Screening

Table 3 shows the associations between demographic, stressor, and cultural buffer variables and odds of lifetime STD screening. The models were fit in a sequential manner, starting with all demographic and socioeconomic variables, then adding stressors (such as Historical Loss Scale and global discrimination scale), finally adding buffer variables (such as preferred spoken language, participating in ceremonies and Spiritual Coping Scale).

**Table 3:** Logistic regression models for STD testing among American Indians with binge substance use in a rural community.

	**Model 1**	**Model 2**	**Model 3**	**Model 4**	**Model 5**
**Covariate**		Odds ratio [95% confidence interval]			
**Sociodemographic characteristics**					
**Age (piece-wise continuous, per year)**					
<26	1.04	1.044	1.03	1.004	1.071
	[0.842, 1.283]	[0.846,1.289]	[0.832, 1.274]	[0.800, 1.260]	[0.840, 1.367]
26– <43	1.07	1.069	1.088^*^	1.104^*^	1.096
	[0.997, 1.148]	[0.995, 1.148]	[1.008, 1.173]	[1.018, 1.197]	[1.000, 1.201]
43+	0.915	0.92	0.911	0.918	0.934
	[0.778, 1.076]	[0.782, 1.082]	[0.771, 1.078]	[0.780, 1.082]	[0.777, 1.122]
**Sex**					
Female vs. male	1.552	1.58	1.515	1.509	1.219
	[0.795, 3.028]	[0.797, 3.131]	[0.756, 3.034]	[0.755, 3.016]	[0.558, 2.666]
**Marital status**					
Married vs. single	0.628	0.638	0.642	0.655	0.488
	[0.320, 1.234]	[0.323, 1.261]	[0.319, 1.294]	[0.321, 1.337]	[0.215, 1.105]
Missing vs. single	0.604	0.595	0.571	0.528	0.468
	[0.146, 2.491]	[0.139, 2.548]	[0.122, 2.677]	[0.121, 2.306]	[0.0930, 2.354]
**Have any children**					
Yes vs. no	1.598	1.578	1.526	1.523	1.787
	[0.773, 3.305]	[0.759, 3.278]	[0.715, 3.257]	[0.690, 3.359]	[0.785, 4.070]
**Education status**					
Holds high school diploma/GED vs. < high school	1.282	1.266	1.298	1.162	1.064
	[0.664, 2.477]	[0.647, 2.477]	[0.657, 2.564]	[0.579, 2.328]	[0.528, 2.143]
**Household size**					
Number of people living in the house (per person)	1.216^**^	1.217^**^	1.204^**^	1.203^**^	1.198^**^
	[1.064, 1.389]	[1.063, 1.392]	[1.053, 1.377]	[1.048, 1.382]	[1.044, 1.375]
**Life stressors**					
**Economic stressor**					
Employed vs. unemployed	0.737	0.731	0.841	1.011	0.775
	[0.256, 2.122]	[0.253, 2.107]	[0.302, 2.344]	[0.366, 2.790]	[0.264, 2.278]
**Traumatic stressors**					
**Historical loss scale**					
Score ≥3 vs. score <3		1.334	1.65	1.947	1.617
		[0.527, 3.372]	[0.598, 4.554]	[0.695, 5.451]	[0.560, 4.663]
Missing score vs. score <3		0.941	0.603	0.378	0.714
		[0.161, 5.508]	[0.0579, 6.274]	[0.0326, 4.395]	[0.0645, 7.909]
**Global discrimination scale**					
Score ≥1 vs. score <1			0.48	0.452	0.401^*^
			[0.222, 1.040]	[0.204, 1.003]	[0.175, 0.921]
Missing score vs. score <1			2.185	2.357	2.068
			[0.176, 27.14]	[0.269, 20.63]	[0.238, 18.00]
**Cultural buffers**					
**Preferred language spoken**					
English vs. native language				1.956	2.076
				[0.801, 4.777]	[0.802, 5.372]
Both english and native language vs. only native language				1.692	1.714
				[0.697, 4.111]	[0.654, 4.497]
Missing vs. only native language				6.228	3.866
				[0.315, 123.1]	[0.304, 49.14]
**Importance of participating in tribe's traditional practices and ceremonies**					
Not very vs. not at all					1.726
					[0.497, 5.992]
Somewhat vs. not at all					1.221
					[0.472, 3.159]
Very important vs. not at all					1.441
					[0.504, 4.124]
Refuse to answer/missing vs. not at all					2.683
					[0.435, 16.57]
**Spiritual coping scale: extent to which spirituality is involved in dealing with stressful situations**					
Not very vs. not at all					0.533
					[0.127, 2.243]
Somewhat vs. not at all					1.409
					[0.448, 4.430]
Very vs. not at all					1.051
					[0.251, 4.413]
Don't know vs. not at all					0.409
					[0.110, 1.516]
Refuse to answer vs. not at all					0.275^*^
					[0.076, 0.987]
*N*	236	236	236	236	236
*AIC*	252.8	256.5	256.5	258.8	266.1

*Exponentiated coefficients; 95% confidence intervals in brackets*.

* *p < 0.05,*

** *p < 0.01*.

*Of 254 participants, 18 are excluded in these models due to missing data on having children (n = 1), education status (n = 2), employment status (n = 2) and number of people in the household (n−14)*.

Among the demographic variables, we found that the number of people living in the household was statistically associated with the outcome of ever screening for STD. Controlling for other demographic variables in the model, for each additional person in the household, estimated odds of STD testing went up by 22% [adjusted OR (aOR) = 1.22, 95%CI: 1.06, 1.39]. The relationship with age was non-linear, with the strongest positive slope for respondents between 26 and 43 years of age. In this group, for every additional year, the odds of ever completing STD testing went up by an estimated 7% (aOR = 1.07, 95%CI: 0.99, 1.15). For those below 26 years of age, the relationship between age and lifetime STD testing was still positive, but did not reach statistical significance. After 43 years of age, there was no positive association between age and lifetime STD testing. These findings were consistent across all models presented in Table 3.

Economic stress did not have a significant association with STD testing in all models. In the full model, the odds of STD testing were 27% lower (aOR = 0.78, 95%CI: 0.26, 2.28) for those experiencing economic stress, but were not statistically significant.

Models 2 through 5 included two AI-specific stressors, namely, the Historical Loss Scale and global discrimination scale, in addition to the demographic variables and economic stress. Greater historical loss score was not statistically significantly associated with higher odds of STD testing in all models. In the final model, those who scored 3 or greater in HLS were 62 percent more likely than those who scored <3 in HLS to get STD testing (aOR = 1.62, 95%CI: 0.56–4.67).

Global discrimination was negatively associated with STD screening in the fully adjusted model. The adjusted odds ratio of lifetime STD testing for those experiencing higher discrimination was 0.40 (95%CI: 0.18–0.92).

Models 4 and 5 included AI-specific cultural buffer variables, such as preferred language to speak at home, level of importance in participation in traditional ceremonies and the Spiritual Coping variable. None of these variables were statistically significantly associated with STD testing after adjustment for demographics and AI-specific stressors.

## Discussion

### Summary of Findings

A primary goal of study was to explore how risk and protective factors from the Indigenist Stress Coping framework predict the behavior of STD screening among AIs with recent binge alcohol use–a high risk population for STDs and HIV. This hard to reach group which is often mobile is an important subgroup in curbing the rising rates of STIs and HIV as binge substance use is often associated with sexual risk taking and poor mental health (59–62). As with HIV cascade of care, testing is the first necessary step for early detection, treatment, and prevention of STD infection (63); and considering STD testing as a positive sexual health behavior allows for advancing resiliency and strength-based approaches. Our results confirmed one of two hypotheses. More specifically, results show that stressor exposures such as economic stress, thoughts of historical loss, and perceived or experienced discrimination specifically decrease the protective sexual health behavior of STD screening.

The majority (76.7%) of participants reported having completed STD screening at least once in their life (*ever tested for STD*). The high self-reported STD testing rate at the baseline assessment was surprising, given the aforementioned screening access barriers in this and other rural and reservation-based tribal communities such as lack of culturally tailored evidence-based STD reduction programs and poor access to clinic or non-clinic-based self-administered STD screening (17, 27). High proportions of lifetime STD testing, however, may not necessarily reflect timely or regular screening among high risk AI with binge substance use. Specifically, it also does not mean that participants were meeting the CDC recommendations for sexually active adults of completing STD testing at least once a year (64). It also does not reflect linkage to care, retention, treatment initiation or adherence (56).

Results also show that number of members in the household was positively associated with STD testing, with the aOR of lifetime STD screening being statistically positively associated in all five models. A published article from this same AI study sample shows that living with an older generation positively predicted intervention retention (55). In this present paper, larger household size may be a protective factor in that traditional AI family systems function as a form of social support. The extended family may also help motivate AIs to take care of their sexual health in the presence of co-occurring substance use and/or facilitate access to care by provision of transportation, appointment reminders, etc. This finding again supports the notion that programming for Native populations may benefit from starting with a collective view or utilizing a framework that includes family and community in addition to the individual.

Supporting our first hypothesis, the findings revealed that perceived discrimination stressors are significant predictors of STD testing. Native adults in this study with fewer discrimination encounters had higher odds of lifetime STD screening. Similarly, those who thought of historical loss less frequently were more likely to have screened for STD at some point in their life. Causal association between historical, intergenerational, and lifetime trauma experiences, discrimination experiences, other stressors, and AI/AN psychological health (e.g., depression, anxiety, post traumatic disorder) have been well-established (44, 46, 65, 66). A recent study in Canada examining the mechanisms of these associations found that discrimination played a mediating role in that the more types of traumatic events Indigenous peoples experienced the greater their perceptions of discrimination stressors, resulting in positive association with depressive symptoms (67). While the present paper cannot establish causal association, it expands the AI/AN specific literature by demonstrating associations between historical loss, discrimination, and STD screening and validates these constructs from the Indigenist Stress Coping framework for predicting a sexual health outcome.

The nature and process of clinic-based STD screening tests necessitate accessing and engaging with health services and health care providers. Many concerns with clinic-based services in Native communities have been cited, including mistrust of health services, discrimination and judgement by healthcare providers, fear of disclosure, and lack of anonymity in small communities where everybody knows each other (63, 66). These concerns may preclude seeking of clinic-based services including STD testing. In a recent study conducted with Native American adults, almost one in six (15%) report they avoided seeking health care for themselves or family members due to anticipated discrimination at the clinic (68). Our findings, while unable to draw causal links, may indicate that those who are more likely to anticipate discrimination in clinic-based settings may be more hesitant to seek out clinic-based STD testing. Thus, we conclude additional efforts to address the aforementioned concerns with clinic-based services (i.e., discrimination and judgement by healthcare providers) for Native populations and/or the provision of non-clinic-based testing services in Native communities (i.e., community-based, self-administered testing) is warranted.

Our second hypothesis which articulated that cultural, community, spiritual, and personal resources/strengths from the Indigenist Stress Coping framework may buffer the effects of stressors on poor sexual health outcomes was not supported by the results from this analysis (31). Even though the directions of the associations between these protective factors were positively associated with lifetime STD screening, they were not statistically significant. It may be that because the idea of, action and experience surrounding STD screening procedures are based on strong western medicine and western science perspectives that cultural connectedness plays a less important role in this behavior. Alternatively, the standard measures employed to assess AI enculturation and spirituality may not be the best fit to predict STD screening, as these measures have found to better predict psychological distress and other mental health symptoms (42, 69). Third, in the context of diminished encounters with historical loss and discrimination associated stressors, cultural factors may not operate as direct buffers to cope with these stressors. Finally, participants in this study who all had recent binge substance use, may have faced other challenges and barriers to engaging in traditional practices and ceremonies; thus, results may underestimate the potential for these protective factors to buffer against risks for poor sexual health outcomes.

### Limitations

These findings should be considered with several limitations in mind. The study sample constitutes a group of community members who had co-occurring binge substance use and were experiencing crisis with regards to need for services, support and care. Therefore, the findings are not generalizable to all AI populations.

Secondly, recall of events or experiences when reporting on the survey could have been incomplete, resulting in missing data, especially as participants had recent binge substance use. The ISC framework-articulated AI historical loss and discrimination stressors and AI cultural buffer variables had sizeable missing data. These series of questions were located at the very end of the baseline assessment questionnaire, and right after a 53-item measure assessing depression, anxiety, and other symptoms of mental health conditions. The participants may likely have experienced respondent fatigue when they got to this point on the survey, and may have wanted to complete or end the survey quickly. Further exploration revealed that the theoretical predictors of interest—AI enculturation and Spiritual Coping Scale in particular—had considerable “Don't know” or “Refuse to answer” responses. Had these sets of questions been placed near the beginning of the survey, participants may have had responded with more time, potentially avoiding missing data.

A little over half the participants reported having none to minimal experiences with discrimination. For reservation-dwelling community members, the probability of discrimination encounters is more likely to occur in surrounding towns off the reservation. The assessment was administered on the reservation, and the survey items on discrimination did not specify the location of discrimination experiences, potentially leading to under reporting of such experiences. Historical loss instrument responses likely had similar issues. Finally, analyses were conducted cross-sectionally and cannot lead to any conclusions about causation between ICS variables and the behavior of STD screening.

### Strengths, Conclusions and Implications

Health disparities in AI/ANs are driven by a complex interplay of historical and lifetime cultural trauma, socioeconomic and environmental inequities, and contemporary socio-cultural and institutionalized discrimination (70, 71). The burden of STD prevalence, incidence, and outcomes is experienced at a disproportionately high rate by AI/ANs. The historical and contemporary Indigenous social determinants of health are multi-level contextual barriers that affect differential AI/AN access to sexual and reproductive health care services including effective interventions to prevent, diagnose, and treat STDs. The STI-National Strategic Plan 2021–2025 articulates the need for integration of alcohol/substance use and STDs services as a promising strategy to promote STD prevention and management among key subgroups, such as the AI sample in this study with recent binge substance use. Our findings suggest that such programs should take into consideration AI-specific historical traumatic stressors such as historical loss, and lifetime discrimination encounters and how these interact to drive or discourage sexual health services at local clinics. Our findings also highlight that these programs consider the extended family as a protective factor in that traditional AI family systems function as a form of social support. Future research should consider improvement in measurements of AI enculturation constructs and data collection minimizing the potential missing data on these variables (42, 69). Future research should also consider the AI/AN experience in STD testing and ensuing STD cascade of care (72, 73) for improving sexual health equity in this priority population.

## Data Availability Statement

The datasets presented in this article are not readily available because Tribal data sovereignty governs the release of and access to the dataset used for this study. Requests to access the datasets should be directed to LT, ltingey1@jhu.edu

## Ethics Statement

The studies involving human participants were reviewed and approved by John Hopkins University Bloomberg School of Public Health IRB, participating Tribal Council, and Tribal Health Advisory Board. The patients/participants provided their written informed consent to participate in this study.

## Author Contributions

MM drafted the manuscript. MM, LT, and RC revised and edited the final manuscript. RC drafted sections of the methods and discussion sections. GY completed all formal data analysis and contributed to the results section. SR contributed to the methods section. MW contributed to the editing. AS, FL, AL, and LP contributed to reviewing and contextualizing research questions and findings interpretation. All authors contributed to the concept of the manuscript, reviewed and edited multiple drafts of the manuscript, read and agreed to the published version of the manuscript.

## Funding

Research reported in this publication was supported by the National Institute of General Medical Sciences of the National Institutes of Health under Award Number U261IHS0093, partly by the National Institute of Mental Health of the National Institutes of Health under Award Number R25MH084565, and by the University of Washington's Center for American Indian and Indigenous Studies (CAIIS) under Special Research Grant Award for open access publication fees.

## Author Disclaimer

The content is solely the responsibility of the authors and does not necessarily represent the official views of the National Institutes of Health.

## Conflict of Interest

The authors declare that the research was conducted in the absence of any commercial or financial relationships that could be construed as a potential conflict of interest.

## Publisher's Note

All claims expressed in this article are solely those of the authors and do not necessarily represent those of their affiliated organizations, or those of the publisher, the editors and the reviewers. Any product that may be evaluated in this article, or claim that may be made by its manufacturer, is not guaranteed or endorsed by the publisher.
